# Heterologous Expression and Biochemical Characterisation of Fourteen Esterases from *Helicoverpa armigera*

**DOI:** 10.1371/journal.pone.0065951

**Published:** 2013-06-17

**Authors:** Mark G. Teese, Claire A. Farnsworth, Yongqiang Li, Chris W. Coppin, Alan L. Devonshire, Colin Scott, Peter East, Robyn J. Russell, John G. Oakeshott

**Affiliations:** 1 CSIRO Ecosystem Sciences, Canberra, Australia; 2 School of Chemistry, Australian National University, Canberra, Australia; 3 School of Biological Sciences, Australian National University, Canberra, Australia; 4 Research and Development Centre of Biorational Pesticides, College of Plant Protection, Northwest A&F University, Yangling, People’s Republic of China; University of Crete, Greece

## Abstract

Esterases have recurrently been implicated in insecticide resistance in *Helicoverpa armigera* but little is known about the underlying molecular mechanisms. We used a baculovirus system to express 14 of 30 full-length esterase genes so far identified from midgut cDNA libraries of this species. All 14 produced esterase isozymes after native PAGE and the isozymes for seven of them migrated to two regions of the gel previously associated with both organophosphate and pyrethroid resistance in various strains. Thirteen of the enzymes obtained in sufficient yield for further analysis all showed tight binding to organophosphates and low but measurable organophosphate hydrolase activity. However there was no clear difference in activity between the isozymes from regions associated with resistance and those from elsewhere in the zymogram, or between eight of the isozymes from a phylogenetic clade previously associated with resistance in proteomic and quantitative rtPCR experiments and five others not so associated. By contrast, the enzymes differed markedly in their activities against nine pyrethroid isomers and the enzymes with highest activity for the most insecticidal isomers were from regions of the gel and, in some cases, the phylogeny that had previously been associated with pyrethroid resistance. Phospholipase treatment confirmed predictions from sequence analysis that three of the isozymes were GPI anchored. This unusual feature among carboxylesterases has previously been suggested to underpin an association that some authors have noted between esterases and resistance to the Cry1Ac toxin from *Bacillus thuringiensis*. However these three isozymes did not migrate to the zymogram region previously associated with Cry1Ac resistance.

## Introduction

The cotton bollworm *Helicoverpa armigera* has evolved resistance to several classes of chemical insecticides over the last 50 years and some of these resistances remain problematic for control despite the widespread uptake of transgenic cotton varieties expressing various insecticidal Bt (*Bacillus thuringiensis*) toxins [1]–[3]. Although cytochrome P450s are often also involved [4], esterases have recurrently been implicated in metabolic resistance to the major organophosphate (OP) and synthetic pyrethroid (SP) insecticides [1], [5]–[11] and even, in some literature, to the Cry1Ac Bt toxins [12]–[15]. Resistance factors attributed to the esterases vary widely among studies but can be high (>100 fold) for both OPs and SPs.

The evidence from over 30 studies implicating esterases in OP and SP resistance has usually taken the form of the synergism of the insecticides by esterase inhibitors, higher levels of *in vitro* esterase activities with artificial substrates like naphthyl acetate and *p*-nitrophenyl acetate, and more intense staining of certain esterase isozymes in extracts of resistant strains [9]. Notably, several different isozymes have been implicated in the latter for each class of chemistry, with some studies individually implicating up to three different bands and additional differences found between studies [9]–[11]. There may be some overlap between the isozymes implicated in the two sets of resistances but the evidence on this remains equivocal because of the use of multi-resistant strains in some studies, as well as differences in electrophoretic methods between several of them.

The fact that the resistances are so often associated with greater esterase isozyme intensities has led some authors [5], [7], [13] to suggest that the mechanisms may involve enhanced sequestration and, in the case of the OPs and SPs, detoxification of the insecticides, via the overexpression of esterases that can bind and possibly hydrolyse them. There is ample precedent for such mechanisms in the case of OP resistance; in particular the well studied cases of OP resistant aphids and culicine mosquitoes [16], [17] show how heavily over-expressed esterases with tight OP binding capability and some, albeit slow, OP hydrolytic activity can confer high levels of protection. We are unaware of any direct precedents for such a mechanism in the case of SP resistance, although there is strong evidence that some insect esterases can hydrolyse SPs with quite high efficiencies *in vitro* [18]–[20]. In the case of resistance to the Cry1Ac Bt toxin it is suggested that membrane-associated esterases in the larval gut may provide alternate binding sites for the toxin and thereby provide some protection for the target sites for the toxin [13]. Direct empirical evidence for this mechanism is currently scarce but there has been speculation that GPI-anchored esterases in the midgut may be involved [21].

Three recent studies have begun to investigate the mechanisms for some of the esterase-resistance associations in *H. armigera* at a molecular level. An EST and proteomic analysis by Teese *et al*. [21] was able to associate particular esterase EST sequences with certain bands from an insecticide susceptible strain that lay in the same zones of the esterase zymogram that other workers had found to be more intensely staining in OP, SP and Bt resistant strains. Wu *et al*. [10] then applied a similar proteomic approach to an SP resistant strain, finding that a specific isozyme zone that was more intensely staining in this strain was associated with four closely related but paralogous esterase ESTs. They then used quantitative rtPCR to show that all four of the cognate esterase genes were over-expressed by a few to several fold in larval midguts and fat bodies of the resistant strain as compared to a susceptible control strain. A subsequent native Western and proteomic analysis by Han *et al*. [11] showed over-expression of several esterase isozymes from a larger (at least 10 gene) clade (named Clade 1 by Teese *et al*. [21]) containing the four genes identified by Wu *et al.* [10] in both an OP resistant strain and a second SP resistant strain as compared to a susceptible strain. There was some overlap between the esterases over-expressed in these two resistant strains and the four identified by Wu *et al.* [10].

This paper provides baseline biochemical data on possible mechanisms underlying the esterase-based components of the various *H. armigera* resistances. Specifically 14 of the 30 full length esterase ESTs so far described for the species have been expressed in a baculovirus system and their products characterised biochemically. Thirteen produced in sufficient quantity were subject to activity and/or binding assays with OPs, SPs and some other substrates and three were tested empirically for GPI anchoring.

## Materials and Methods

### Esterases Selected for Expression

Fourteen full-length *H. armigera* esterase genes from a total of 30 recovered from a midgut cDNA library [10], [21] were expressed in the baculovirus system. All these genes were from the Australian GR strain, which is fully susceptible to OPs, SPs and the Cry1Ac toxin of Bt (R. Mahon pers. comm.). Eight of them (*CCE001b, 001c, 001d, 001f, 001g, 001h, 001i*, *001j*) were from Clade 1 and these included three of the four (*CCE001d, 001i* and *001j* but not *001a*, which we were unable to express in soluble active form) found to be over–expressed in a Chinese SP resistant strain by Wu *et al*. [10]. The other six (*006a*, *006b*, *014a, 016a, 017a* and *025a*) were selected to represent diverse other clades in the phylogeny produced by Teese *et al.* [21]. Seven of the 14 (*001c*, *001d*, *001i, 001j, 006a*, *017a and 025a*) have been linked previously to specific esterase isozymes in the proteomic experiments of Wu *et al*. [10], Han *et al*. [11] and Teese *et al*. [21]. Twelve of the 14 expressed esterases were predicted by various algorithms to be secreted soluble proteins (most of the Clade 1 enzymes plus 006a, 006b and 025a) or attached to membranes by GPI anchors (001f, 001g, 016a) [21]. The other two were predicted to be intracellular, either cytoplasmic (014a) or transported to the mitochondria (017a).

### Baculovirus Expressions

Gateway-compatible entry vectors were constructed for the untagged expression of all genes of interest, using their native start and stop codons with the addition of an efficient translation initiation sequence immediately 5′ of the start codon. Each of the genes was transferred into linear *Autographa californica* nucleopolyhedrosis virus (AcNPV) DNA (see Fig. S1). The two negative control constructs used were uninfected Sf9 cells and AcNPV-GUS, expressing β glucuronidase [18], [19]. AcNPVs expressing the wild-type and G137D and W251L mutants of the blowfly E3 gene [18], [22] were used as positive controls in some experiments. Transfection of Sf9 cells with AcNPV constructs and preparation of soluble intracellular extracts from high titre stocks were carried out essentially as per Heidari *et al.* [22] and Devonshire *et al.* [23], the major modification being the addition of a second centrifugation step (5,000 g, 60 min) after cell lysis to further clarify the samples. Some other minor modifications for one experiment are noted below.

#### Phosphatidylinositol-specific phospholipase C (PI-PLC) treatment

A modified version of the assay of Incardona and Rosenberry [24] was used to determine whether three enzymes which sequence analysis [21] predicted to contain a GPI anchor (CCE-001f, 001g and 016a) did in fact do so. Sf9 cells were seeded onto polystyrene 6-well plates (Becton-Dickinson, USA) at a density of 2×10^8^ cells per well in 2 ml of Sf900 II serum-free media. After one hour to allow cell attachment, cells were infected with either AcNPV-001g, AcNPV-001f, AcNPV-016a or AcNPV-eGFP at a multiplicity of infection (MOI) of 5. The media was discarded after 48 hours and the cells gently washed with warm phosphate-buffered saline (PBS). The cells were then incubated for 90 minutes in 0.3 ml PBS only, or PBS containing 0.3 U.ml^−1^ phosphatidylinositol-specific phospholipase C (PI-PLC). The liquids were then collected and clarified by centrifugation at 11,000 g for 5 minutes. The adhered cells that remained were also harvested, by adding 0.5 ml of homogenisation buffer (50 mM Tris/Cl pH 8.0, 1 mM EDTA, 0.5% Triton X-100 and 20% w/v sucrose) to each well, followed by mechanical scraping with a pipette tip, and lysis by vortexing. Both the supernatants and cell extracts were stored under sterile conditions for less than a week at 4°C before carboxylesterase and acetylcholinesterase (AChE) activity assays were performed.

In total there were four differences between the transfection and extract preparation procedures in this PI-PLC experiment and those outlined in the previous section for the other experiments herein. For the PI-PLC experiment we used static as opposed to suspended liquid cultures as seed cultures, we transfected at a multiplicity of infection (MOI) of 5 as opposed to 2, harvesting was done at 48 versus about 72 hours and cells were lysed by vortexing rather than repeated passage through a 21 g needle.

### Enzyme Assays

Native polyacrylamide gel electrophoresis (PAGE) and carboxylesterase staining with 1-naphthyl acetate (1-NA) were conducted as described by Han *et al*. [11]. Staining for AChE activity was performed using the method of Li *et al.* [25].

Spectrophotometric assays for carboxylesterase activity against 1-naphthyl acetate were carried out for the PI-PLC experiment above using the methods of Mastropaolo and Yourno [26] adapted to a microplate layout. Reactions were carried out at 28°C in a final volume of 200 µl containing 5–20 µL of enzyme sample, 500 µM 1-naphthyl acetate, 2% *v*/*v* ethanol and 1× PBS in a UV-Star 96-well microplate (Greiner Bio-One, Switzerland). Enzymes were diluted to 100 µL in 1× PBS pre-incubated at 28°C. Immediately prior to commencing the assay, an ethanolic solution of 1-naphthyl acetate was diluted to 1 mM (4% *v*/*v* ethanol) in warm 1× PBS. Reactions were started by adding 100 µl of this substrate solution to the diluted enzyme. Formation of 1-naphthol was monitored by recording the change in absorbance at 235 nm for 10 min in a SpectraMax 190 plate reader (Molecular Devices, USA). Extracts of uninfected Sf9 cells representing equivalent pre-harvest cell densities were used as negative controls in these assays.

The other quantitative enzyme assays below used either uninfected Sf9 cells or AcNPV-Gus constructs as negative controls. For each dilution of each enzyme assayed, a dilution of the control extract representing an equivalent pre-harvest cell density was used and the activity for this control was subtracted from the value for the test enzyme under these conditions. The controls had minimal activities in all the assays below.

Spectrophotometric microplate assays for AChE activity [27], [28] were carried out at 28°C in a final volume of 200 µl containing 20 µL of enzyme sample (2.3×10^−3^ U of acetylcholinesterase from human erythrocytes (Sigma) was used as a positive control), 1 mM 5,5′-dithiobis-(2-nitrobenzoic acid) (DTNB) (Sigma), 0.5 mM acetylthiocholine iodide (Sigma) and 1× PBS in a polystyrene 96-well microplate (Greiner Bio-One). Enzymes were pre-incubated in the microplate at room temperature with DTNB for 20 min, then 28°C for a further 5 min, whereupon the reaction was started by the addition of 20 µl of substrate. Absorbance at 405 nm was monitored for 20 min in a SpectraMax 190 plate reader.

Fluorometric assays for the hydrolysis of the OPs dimethyl 4-methylumbelliferyl phosphate (dMUP, [23]), and diethyl 4-methylumbelliferyl phosphate (dEUP, Sigma) were conducted as previously described [20], [22], [23]. These assays provide measures of substrate turnover rate and the concentration of expressed enzyme active sites (enzyme titre) via the burst equation;
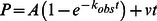
where *P* is the product formed, *A* is the amplitude of the burst phase (enzyme titre), *k_obs_* is the rate constant of the burst phase (ie the rate of substrate binding), *v* is the substrate hydrolysis rate once the enzyme has become saturated and *t* is time [29]. Initial estimates of *A*, *k_obs_* and *v* made as per Coppin *et al*. [20] were refined with the solver function of Excel (Microsoft) so as to minimise the residual sum of squares. The rate of substrate turnover under saturating conditions (kcat [23]) was then calculated as the ratio *v*/A.

Assays for the hydrolysis of SPs – esfenvalerate (Sigma) and the eight individual isomers of cypermethrin – were carried out by monitoring substrate loss by HPLC. The cypermethrin isomers were prepared from technical grade beta-cypermethrin or cypermethrin (both AK Scientific, USA) by the Analytical and Preparative Enantioselective Chromatography Facility, University of Queensland, Australia. A methanolic solution of SP substrate was diluted to 200 µM (1.5% *v*/*v* methanol) in 25 mM Tris-HCl, pH 8.0 and 50 µL of the resulting emulsion was dispensed into separate microfuge tubes for each time point (0, 30, 60 and 120 min) and pre-incubated at 30°C for 5 min. Expressed enzymes were diluted to 0.5% *v*/*v* in pre-warmed (30°C) 25 mM Tris-HCl, pH 8.0. Reactions were started by adding 50 µL of diluted enzyme to each reaction tube and incubating at 30°C. Reactions were stopped by the addition of 100 µL of acetonitrile and vortexing. Samples were then centrifuged at maximum speed in a benchtop microfuge (Eppendorf) for 10 min to sediment protein debris before transferring 150 µL of the supernatant into a 300 µL glass insert within a 2 ml autosampler vial (Agilent, USA). 5 µL of each reaction was injected on an Agilent ZORBAX Eclipse XDB-C18 column (2.1×30 mm, 3.5 µm) using an Agilent 1200 series HPLC. All substrates were separated isocratically with 65∶35 acetonitrile/0.5% acetic acid in water (*v*/*v*) at 1 mL.min^−1^ and monitored at 200 nm. Given the complexities in dealing with substrates of such low aqueous solubility (sub- to low micromolar [18]), results are simply presented as rates of substrate loss rather than estimates of formal kinetic parameters.

## Results

### Native PAGE Analysis of Expressed Proteins

**Figure 1:**
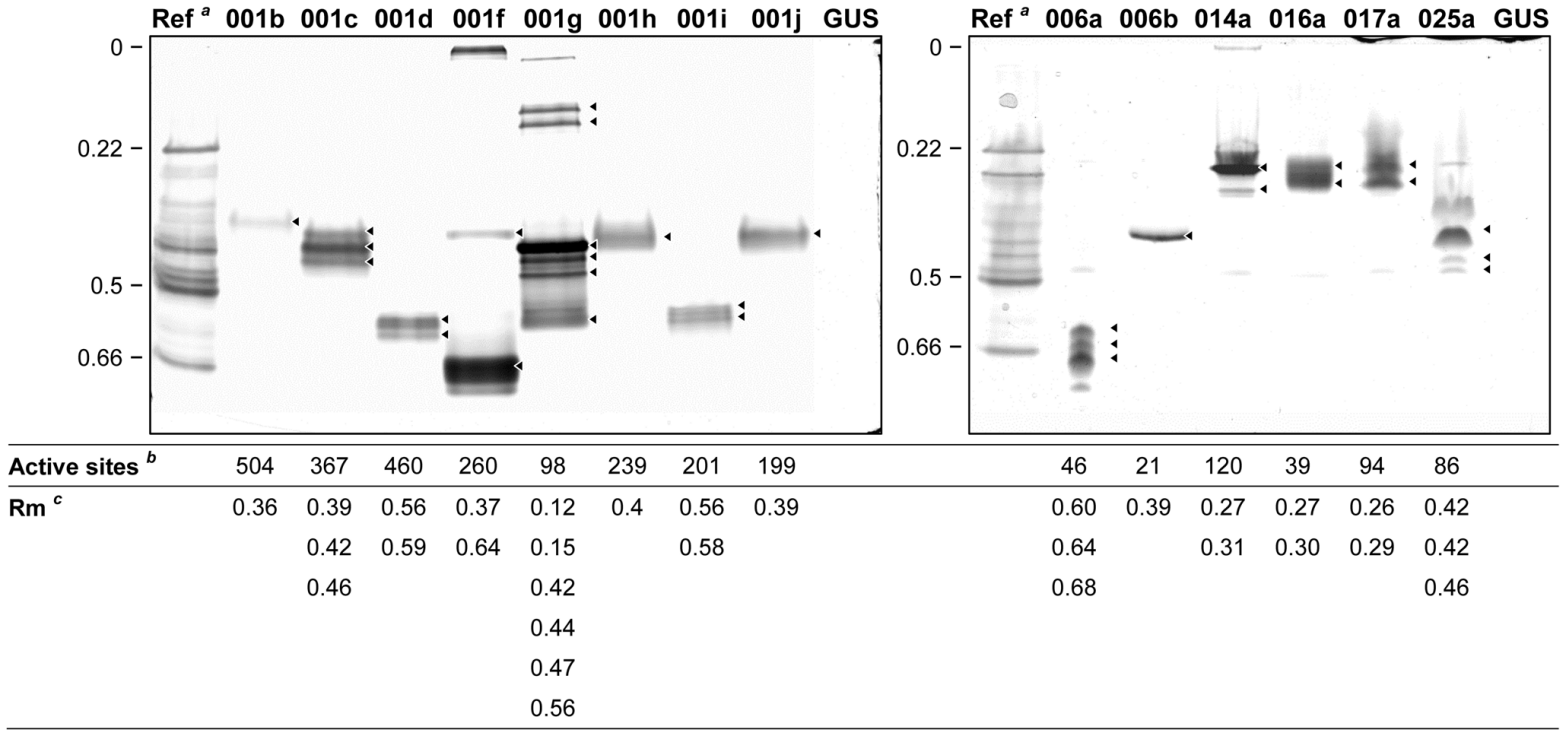
Isozyme profiles of the 14 heterologously expressed esterases on native PAGE. All expressed enzyme samples were derived from the soluble intracellular fraction. Loading volumes were optimised for each sample in order to best display the isozyme pattern, with between 4×10^5^ (014a) and 2.5×10^6^ (001b and 006b) cell equivalents loaded per lane. 10^6^ cell equivalents were loaded in the GUS control lanes (directly comparable to 001c, 006a, 017a and 025a), ^a^ A homogenate of 5th instar larvae (∼15 GR individuals). *^b^* The total number of esterase active sites loaded in each lane as calculated from the titration values (see Table 1). *^c^* R_m_ values of major isozymes (◂) for each expressed esterase.

Native PAGE with carboxylesterase staining revealed distinct isozyme phenotypes for all 14 of the expressed esterases (Fig. 1). Although most of the proteins had been predicted to be secreted (see above), all but one of them were still contained in the soluble intracellular fraction at the time of harvest (∼ three days post infection). The one exception was *006b*, whose product was better visualised in the media (data not shown). In broad terms our results concur with those of Tsubota *et al.* [30] in this respect; they expressed six esterases from the silkworm *Bombyx mori* in the baculovirus/Sf9 system and found most were still associated with the soluble intracellular fraction even though their sequences generally contained secretion signals. This is consistent with the progressive deterioration of the post-translational processing machinery for secreted proteins as virus replication proceeds in the cell.

Four of the 14 esterase genes (*001b, 001h, 001j, 006b*) each produced a single banded isozyme phenotype and six others (*001c, 001d, 001i, 014a, 016a, 017a*) each generated 2–4 closely migrating isozymes (separated by R_m_ values of 0.03 to 0.07) that typically formed a larger zone of activity as staining progressed (Fig. 1). Both these classes of phenotype are common among eukaryote esterases [31], [32]. The other four genes (*001f, 001g, 006a, 025a*) produced multiple bands with a wider range of R_m_ values, albeit each of them produced one dominant band. Previous work on insect esterases has explained multiple closely migrating bands encoded by single genes in terms of alternate glycosylation states for secreted enzymes [31], while more distantly migrating isozymes from single genes have been variously attributed to monomer/dimer differences [33], radical post-translational effects such as GPI anchoring [34] and, very occasionally, alternate start or splice sites [30]. As noted above most of our esterases have secretion signals and all that do have at least two consensus *N*-linked glycosylation sites [21]. Teese *et al*. [21] also found three of the esterases we have expressed have consensus sequences for GPI anchoring (001f, 001g, 016a; see above). Tsubota *et al.* [30] also found that the *B. mori* orthologue of *014a* was subject to both alternate start sites and alternate splicing.

Seven of the genes studied here had also been matched to larval isozymes in the proteomic experiments of Wu *et al.* [10], Han *et al.* [11] and Teese *et al.* [21] and there was good agreement between the phenotypes of the baculovirus products and the proteomics for four of these (*001c, 006a*, *017a* and *025a*; Fig. S2). The differences for the other three, *001d*, *001i* and *001j*, could be due to genetic polymorphism, since the genes for the current work come from an Australian strain and Wu *et al*. [10] and Han *et al*. [11] studied Chinese strains, or to incomplete recovery of bands in the proteomics in the case of Teese *et al*. [21] (who only extracted part of the zymogram profile and therefore may have missed the major products of some genes).

### Testing for GPI-anchors in Three of the Esterases

Teese *et al*. [21] proposed that some fraction of the enzyme produced by four *H. armigera* esterase genes (*001f*, *001g*, *002a* and *016a*) could be anchored to the surface of the cell membrane by a GPI-anchor because they contained consensus hydrophobic C-terminal recognition sequences for cleavage of the protein prior to attachment of the glycophospholipid anchor. Three of the putatively GPI anchored esterases, 001f, 001g and 016a, were expressed in this study; 002a lacks all three ‘catalytic triad’ residue(s) and is assumed to be catalytically inactive [21]. Two of the three we have expressed (001f and 001g) were amongst those showing relatively complex isozyme profiles on native PAGE that could at least in part reflect different steps in the anchoring process (see Fig. 1 and above).

**Figure 2:**
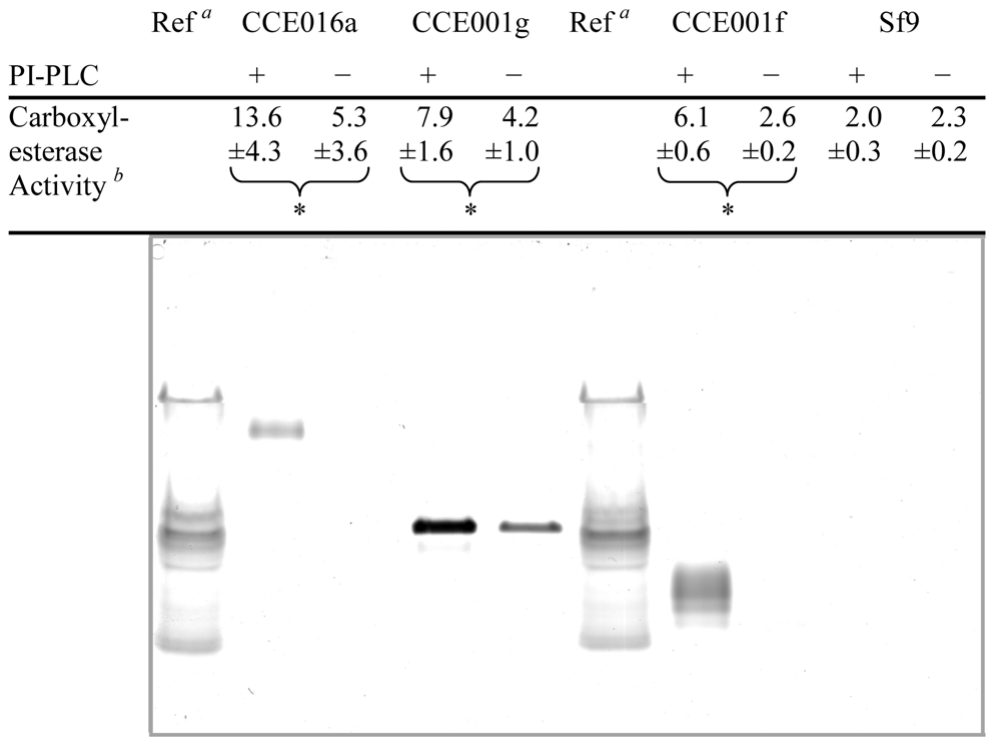
PI-PLC-mediated release of esterases from Sf9 cells expressing 016a, 001g and 001f. Cells expressing the respective enzymes were incubated with PI-PLC (+) or buffer only (−), and the carboxylesterase activity associated with the supernatant was measured (1-NA substrate). For each of the expressed esterases, PI-PLC treatment significantly increased the amount of soluble carboxylesterase activity compared to the respective buffer only treatment (indicated with an asterisk - see text), but did not affect the soluble esterase activity of Sf9 control cells. One representative sample (15 µl) of each treatment was analysed by native PAGE. No bands were apparent in the Sf9 control cells (or expressing GFP – results not shown), however in cells expressing *H. armigera* esterase, PI-PLC treatment increased the staining of the (most abundant) isozymes previously associated with these enzymes (Figure 1). *^a^* As per Fig 1. *^b^* Specific activity towards 1-NA (M.min^−1^.g^−1^ total protein) and standard deviations based on three biological replicates.

The diagnostic test for a GPI-anchored protein involves treatment with a specific phospholipase C (PI-PLC) [35]. This assay has been previously used to confirm the GPI-anchor of AChE from *Drosophila melanogaster* [24]. We have found that PI-PLC treatment of Sf9 cells expressing *001f*, *001g* and *016a* results in significant solubilisation of carboxylesterase activity (t_4_ = 43.54, P<0.001, t_4_ = 5.87, P = 0.014 and t_4_ = 3.80, P = 0.031 respectively) and increased staining of isozymes at the appropriate R_m_ values (Fig. 2). These data provide strong evidence that a proportion of each of these three esterases is GPI-anchored. Spectrophotometric assays showed none of 001f, 001g and 016a to have any detectable activity towards the artificial AChE substrate, acetylthiocholine (data not shown), suggesting that the recombinant enzymes would not be efficient AChE alternatives.

Interestingly, the untreated 001g extracts in this experiment did not show the multiplicity of isozyme bands evident in the original electrophoretic comparisons in Figure 1, albeit those for 001f and 016a did so. We suspect that minor differences between the experiments in the transfection and extract preparation procedures (eg in MOIs and incubation times; see Materials and Methods) would have led to differences in the degree of virus replication and hence in the disruption to post-translational processing. This could in turn lead to differences in isozyme profiles. We do not know why such differences occurred for 001g but not the other two enzymes; further work will be required to elucidate the details of the processing of these three GPI-anchored esterases.

### Associations with Isozymes Previously Associated with Resistance

**Figure 3:**
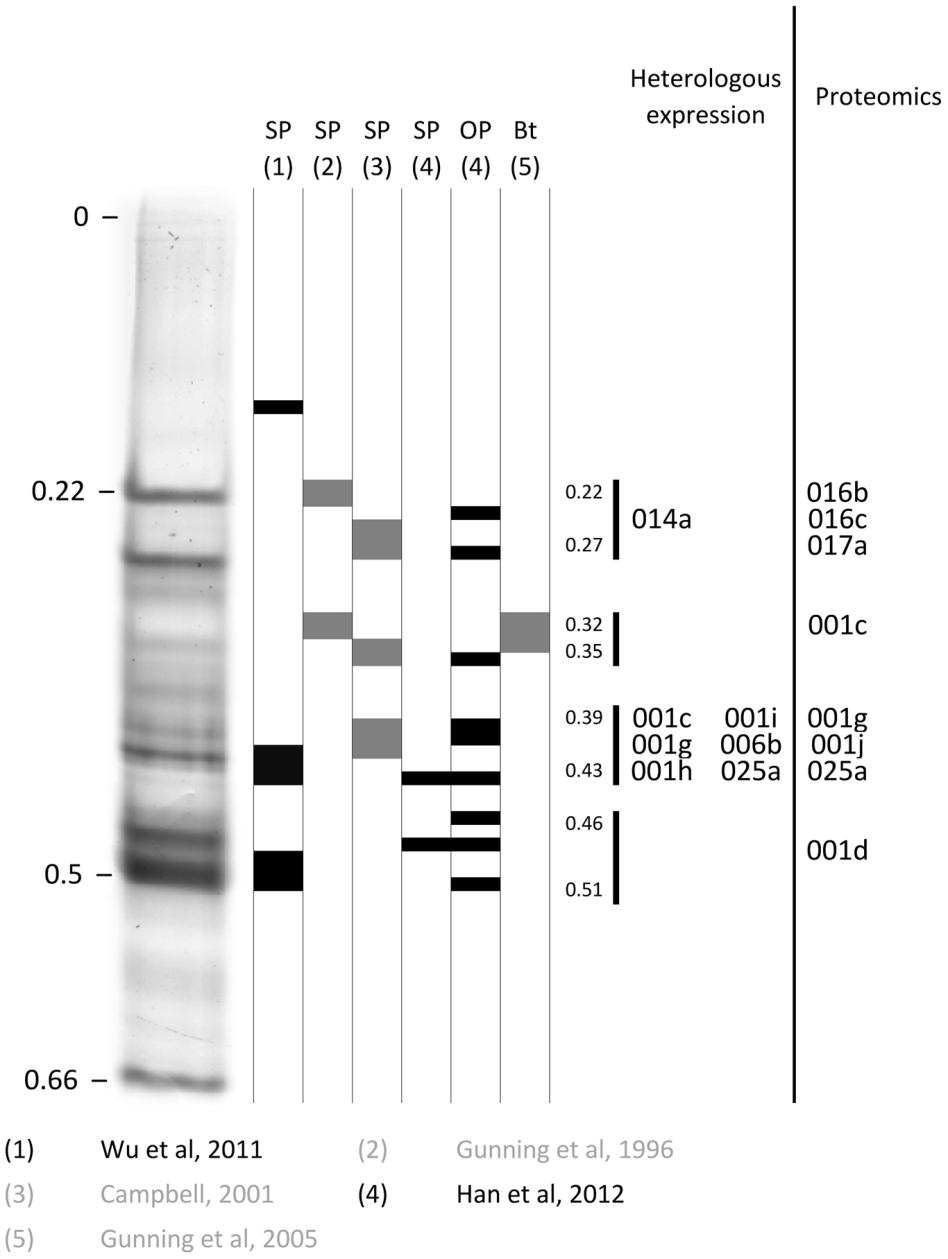
Comparison of esterase isozymes previously associated with OP, SP or Bt resistance with those expressed herein and those previously identified by proteomics. On the left is a lane of a native PAGE gel stained for esterase activity loaded with a mass homogenate of fifth instar *H. armigera* larvae of the insecticide susceptible GR strain. In the centre of the figure are diagrammatic representations of regions of activity associated with resistance in strains from China (black) and Australia (grey). The R_m_ values for four zones of activity recurrently implicated are then indicated, with the specific esterases implicated in these zones by the heterologous expressions herein or previous proteomic studies on the right.

Several studies have previously associated resistance to OPs, SPs and less commonly to the Cry1Ac Bt toxin with more intense staining of particular esterase isozymes (see Introduction). Isozymes with a wide range of mobilities across the isozyme profile have been implicated. Most of the studies have used different electrophoretic methods from those used herein, so precise comparisons with the R_m_ values of our heterologously expressed enzymes are not possible in those cases. However the work of Han *et al*. [11] was based on precisely the same electrophoretic methods as herein and we have also calibrated the results of Wu *et al*. [10] against our methods by running their extracts under our conditions [11]. Moreover we find that the methods of Gunning *et al*. [5], [13], and Campbell [36] generate broadly similar electrophoretic profiles and R_m_ values (within ∼0.03 for most of the profile) to ours, albeit we were not able to use precisely the same extracts or strains in these comparisons (data not shown). Fig. 3 therefore compares the Rm values from Gunning *et al*. 5,13, Wu *et al*. [10], Han *et al*. [11] and Campbell [36] to those of the major isozymes produced by our 14 heterologously expressed esterase genes.

Fig. 3 shows that OP and SP resistances have each been associated with four major regions (R_m_ 0.22–0.27, 0.32–0.35, 0.39–0.43 and 0.46–0.51), with Cry1Ac resistance also associated with the second of these (0.32–0.35). Of particular significance to the current study, the first region (0.22–0.27) includes the 014a enzyme expressed herein (as well as proteomic matches to 016a, 016b and 017a), while the third region (0.39–0.43) includes the 001c, 001g, 001h, 001i, 006b and 025a expressed herein (plus proteomic matches to 001g, 001j and 025a). However, none of the enzymes expressed herein (and only the one proteomic match – to 001c) corresponds to the second region, associated with Cry1Ac resistance. It is notable that none of the three GPI anchored proteins 001f, 001g and 016a expressed herein corresponds to this region, since the expectation of Teese *et al*. [21] was that the esterases associated with Cry1Ac resistance would be GPI anchored.

### OP and SP Hydrolase Activities

We have determined the activities of 13 of the heterologously expressed enzymes for two model OPs, eight resolved isomers of the SP cypermethrin and the major insecticidal isomer of another SP, fenvalerate. Too little enzyme was available in the 006b extracts to include them in these analyses.

All 13 of the enzymes tested had low but measurable activities against the diethyl OP dEUP and the dimethyl OP dMUP (Table 1). Several had activities that were at least two fold higher than the wild-type E3 enzyme from OP susceptible *L. cuprina.* However none had activities approaching the values associated with the two mutant E3 enzymes that confer resistance on that species (W251L associated with resistance to dimethyl OPs and G137D associated with resistance to both diethyl and dimethyl OPs (Table 1) [22], [23], [37], [38]. As noted above, the GR strain from which our *H. armigera* esterase genes were obtained is susceptible to OPs. The eight esterases from the Clade (1) which Wu *et al*. [10] and Han *et al*. [11] had associated with xenobiotic resistance did not yield higher OP hydrolase activities as a group than did those from the other Clades tested, although some Clade 1 enzymes (eg 001g, 001h and 001j) in the third zymogram region associated with OP resistance in the previous section did yield values in the higher range of those obtained.

**Table 1:** Titration values and turnover rates (*k_cat_*) for the two OPs dEUP and dMUP for 13 expressed *H. armigera* esterases and the three E3 control enzymes.

Enzyme	Titre (µM)		*k_cat_* (dEUP)(min^−1^.10^3^)		*k_cat_* (dMUP)(min^−1^.10^3^)	
001b	3.2	(0.4)	24.6	(2.9)	15.5	(0.6)
001c	4.3	(2.0)	13.0	(2.5)	26.5	(2.7)
001d	3.6	(1.3)	9.4	(1.6)	11.4	(1.2)
001f	8.6	(1.1)	14.0	(2.0)	16.6	(3.2)
001g	1.4	(0.5)	21.9	(1.7)	27.9	(1.8)
001h	2.7	(0.3)	19.3	(0.7)	27.2	(3.8)
001i	1.6	(0.4)	19.0	(3.5)	15.5	(2.1)
001j	2.2	(0.1)	13.3	(1.1)	29.7	(2.2)
006a	1.1	(0.2)	14.0	(0.8)	11.7	(2.9)
014a	4.2	(1.1)	18.3	(0.7)	21.7	(1.1)
016a	0.7	(0.2)	18.1	(3.0)	22.9	(2.7)
017a	2.8	(1.5)	3.0	(0.2)	6.3	(1.2)
025a	1.2	(0.4)	9.3	(1.4)	8.4	(2.2)
E3 WT	4.2	(3.0)	8.8	(1.0)	13.3	(0.7)
E3 W251L	1.3	(0.3)	27.5	(3.3)	182.9	(7.3)
E3 G137D	1.5	(0.4)	92.4	(3.7)	144.3	(6.8)

The data for each enzyme are based on an average of two biological replicates (different expressions) and six technical replicates (different assays). All data are given as means with standard errors. The titration values are taken from the dEUP data, however there was no significant difference between the estimates from the dEUP and dMUP data sets. The enzymes are ordered according to their phylogenetic relationships as presented in Wu *et al.* [10] and Teese *et al*. [21].

Notably one of the latter (001h) also bore the equivalent of the W251L substitution that enhances the OP hydrolase activity of *L. cuprina* E3. However two others in our data set which also bore this substitution (001c and 001i) did not show notably high OP hydrolase activities. By comparison, Cui *et al*. [39] found that seven of the eight insect esterases they mutated *in vitro* to carry this substitution had at least somewhat higher OP hydrolase activities than the corresponding wild-type enzymes.

In general terms the expressed *H. armigera* esterases show considerably (often ∼ three logs) higher activity against esfenvalerate and the eight cypermethrin isomers than against the OPs (Table 2). However, the enzymes differ markedly in their relative activities across the nine SP isomers tested; no enzyme shows relatively high activity against all nine, and those with relatively high activity for particular isomers differ considerably among the nine. Notably also, there is significant activity for each of the three most insecticidal isomers, with 016a most effective against 1*(R)trans-α(S)* cypermethrin, this enzyme and 017a most effective against 1*(R)cis-α(S)* cypermethrin, and this enzyme plus 001g most effective against esfenvalerate (ie 2*(S)-α(S)* fenvalerate). Enzymes from Clades 16 and 17 were included in the first region associated with SP resistance in the preceding Section, and 001g was included in the third such region.

**Table 2:** Rates for the hydrolysis of the eight isomers of cypermethrin and esfenvalerate by 13 expressed *H. armigera* esterases and two E3 control enzymes.

Enzyme	Cypermethrin isomers																Esfenvalerate	
	1(*S*)*trans*-α(*S*)		1(*S*)*trans*-α(*R*)		1(*R*)*trans*-α(*S*)		1(*R*)*trans*-α(*R*)		1(*S*)*cis*-α(*S*)		1(*S*)*cis*-α(*R*)		1(*R*)*cis*-α(*S*)		1(*R*)*cis*-α(*R*)		2(*S*)-α(*S*)	
001b	37.3	(1.6)	32.3	(2.0)	4.4	(1.7)	7.9	(0.5)	2.1	(0.3)	5.8	(3.6)	3.9	(3.9)	5.4	(1.5)	1.5	(1.5)
001c	36.2	(14.3)	40.4	(6.3)	12.5	(3.5)	1.4	(1.2)	6.6	(6.2)	1.5	(0.9)	1.9	(0.8)	2.4	(1.6)	1.2	(1.2)
001d	16.0	(3.6)	24.6	(4.4)	6.2	(3.7)	3.2	(0.9)	10.1	(9.9)	1.9	(0.9)	4.1	(2.7)	3.4	(1.4)	0.8	(0.5)
001f	3.0	(0.2)	2.8	(0.2)	0.0	(0.0)	0.4	(0.0)	0.2	(0.2)	0.9	(0.9)	1.3	(1.3)	2.7	(0.9)	0.0	(0.0)
001g	12.9	(6.7)	12.8	(2.2)	0.0	(0.0)	8.8	(8.8)	1.3	(1.3)	15.7	(0.3)	9.3	(9.3)	23.2	(12.3)	17.0	(11.4)
001h	5.9	(2.9)	0.6	(0.6)	0.0	(0.0)	3.2	(3.2)	0.0	(0.0)	1.6	(1.6)	10.5	(2.6)	9.1	(9.1)	0.0	(0.0)
001i	2.8	(1.1)	0.0	(0.0)	7.9	(3.0)	2.9	(1.7)	4.7	(4.7)	2.5	(1.6)	4.5	(2.1)	12.3	(4.6)	5.4	(3.6)
001j	32.9	(2.9)	55.1	(3.9)	0.5	(0.5)	9.1	(3.6)	2.2	(1.1)	9.0	(3.5)	11.6	(3.0)	12.4	(1.9)	1.8	(1.8)
006a	0.0	(0.0)	3.0	(3.0)	3.6	(2.1)	0.4	(0.4)	11.8	(11.8)	5.0	(5.0)	7.4	(2.6)	0.0	(0.0)	8.2	(8.2)
014a	2.1	(2.1)	1.7	(1.5)	13.7	(1.6)	3.5	(1.7)	13.1	(6.0)	3.6	(3.6)	9.2	(1.3)	4.4	(2.0)	14.9	(2.6)
016a	0.0	(0.0)	16.0	(16.0)	33.8	(17.7)	3.9	(0.1)	3.2	(3.2)	56.6	(25.2)	34.6	(17.3)	0.4	(0.4)	17.3	(17.3)
017a	0.0	(0.0)	0.0	(0.0)	13.2	(9.2)	3.5	(3.5)	51.9	(45.4)	11.4	(0.3)	41.1	(34.4)	9.1	(9.1)	4.2	(4.2)
025a	0.0	(0.0)	4.8	(4.8)	2.3	(1.9)	16.2	(14.7)	27.6	(22.5)	9.4	(4.9)	11.6	(4.1)	5.3	(5.3)	7.3	(6.5)
E3 WT	22.3	(2.5)	11.5	(5.9)	1.8	(1.8)	5.7	(5.7)	0.0	(0.0)	14.8	(1.8)	18.5	(18.5)	10.6	(10.6)	1.9	(1.9)
E3 W251L	108.8	(14.5)	28.3	(4.2)	7.5	(5.4)	11.7	(6.6)	4.1	(2.3)	12.1	(7.7)	35.5	(5.8)	20.0	(8.0)	61.4	(26.6)

The values shown are the mol substrate hydrolysed per mol of enzyme per minute under the conditions of the assay. They are means with standard errors based on an average of four replicates. The enzymes are ordered according to their phylogenetic relationships as presented in Wu *et al.* [10] and Teese *et al*. [21]. Note that the large standard errors for many estimates reflect the multiplicative effects of the error variances in the estimates of pyrethroid hydrolysing activities and enzyme concentrations (from Table 1).

There is also a phylogenetic basis for some of the variation among the enzymes in their isomer specificities. For example the 001b/001c/001d/001j subclade all show relatively high activities for the 1*(S)trans-α(S)* and 1*(S)trans-α(R)* pair of cypermethrin isomers, and the distinct but closely related 016a and 017a show relatively high activities for the 1*(S)cis-α(R)* and 1*(R)cis-α(S)* cypermethrin isomers. Of particular note given the RT-PCR evidence of Wu *et al*. [10] for the relatively high expression of esterases from the subclade containing 001b, 001c, 001d and 001j in a fenvalerate selected Chinese line, they all had relatively low levels of esfenvalerate activity – indeed all except 001g of the eight Clade 1 esterases tested had relatively low esfenvalerate activities.

No esterase known to contribute to SP resistance in another species has been characterised biochemically for its SP hydrolytic activity. Indeed the only other insect esterases characterised in this way are variants of the E3 enzyme of *L. cuprina* [18]–[20], two of which were used as positive controls in the SP experiments herein. *L. cuprina* is not generally exposed to SPs and does not show resistance to them [40]. Consistent with the findings of Coppin *et al.* [20], the E3 wild type enzyme shows relatively low activity for most of the isomers tested here, but the W251L variant gives much higher values for two isomers, 1*(S)trans-α(S)* cypermethrin and esfenvalerate, than any of the *H. armigera* esterases tested here. The activity of this enzyme was about 2 and 4 fold higher than that of the best *H. armigera* esterase for those isomers, respectively. There is thus no reason to consider that the SP activities of the *H. armigera* esterases would be unusually high.

## Discussion

The 14 esterases studied here include eight of the ten so far identified from the Clade (1) which had been implicated in OP and SP resistance by proteomic and quantitative rtPCR studies. However these 14 esterases only constitute about half the known midgut-expressed esterases already described from this species [10], [21], with the likelihood that at least some more also remain to be discovered, possibly including in Clade 1. We also note that the enzymes we have expressed are all from an Australian strain susceptible to OPs, SPs and Cry1Ac, and there are quite likely to be allelic differences in the versions of those enzymes associated with the various resistances in the Australian and Chinese stocks studied previously. It is therefore unsurprising that the 14 genes we have expressed only produce isozymes in two of four regions of the *H. armigera* esterase zymogram associated with OP and SP resistance, and none of them produce the isozyme(s) associated with resistance to the Cry1Ac Bt toxin. Notwithstanding these qualifications, however, several important insights emerge from our findings.

We find that all 14 of the esterases could be titrated with our model OPs, meaning that they bound the OPs with relatively high affinity. They also show measurable but low OP hydrolytic activity, generally about two-fold higher than that of the wild type (susceptible) blowfly E3 esterase. However their activities remain several fold lower than the values seen for the two E3 mutants associated with OP resistance in the blowflies. Notably also, there was little difference in activity between the eight esterases from Clade 1 and the five from other Clades. One interpretation of these results could be that OP resistance could be achieved in this species by wholesale over-expression of large numbers of midgut esterases, each of which shows tight OP binding and low but non-negligible OP hydrolase activity.

However, there are also two other viable hypotheses to explain the data to date. One is simply that we have not yet expressed the enzymes that are causally involved in resistance. Another is that the resistance is explained by one or more of the 14 esterases studied here, but at most only partly by the over-expression of these genes. Instead it may be due to amino acid substitution(s) in the version(s) of one or more of these enzymes in OP resistant strains that enhance their OP hydrolase activities. Substitutions corresponding to one of the blowfly E3 esterase mutations that enhance its OP hydrolase activity were in fact found in three of our Clade 1 esterases, but the OP activities of these three enzymes were not qualitatively different from those of our other enzymes. Nevertheless it remains possible that other mutations occur in one or more of these enzymes in the resistant strains which boost their OP activities.

There is a fundamental difference between the OPs and SPs in respect of possible esterase-based metabolic resistance mechanisms. The OPs are hemi-substrates for carboxylesterases and their binding and slow turn-over by esterases *in vivo* can effectively sequester the pesticide [32]. However the SPs are carboxylesters for which there is no evidence for tight binding but slow hydrolysis by esterases [41]. There is in fact much less biochemical data on the interaction of insect esterases with SPs than there is on their interactions with OPs, but the data available suggest that they can have quite high turn-overs for SPs [20]. And, while there is abundant evidence from certain other species (aphids and mosquitoes in particular) that over-expression of appropriately located esterases can confer OP resistance via tight binding/slow turn-over/sequestration 16,17, there is no evidence as yet that establishes causal links between esteratic activities against SPs and the associations between over-expressed esterases and SP resistance in any insect species.

A major reason for the latter has been the isomeric complexity of most SPs and the difficulty of carrying out isomer-specific activity assays. The work herein and that of Coppin *et al*. [20] on various variants of the blowfly E3 esterase are in fact the first comprehensive isomer-specific assays conducted on insect esterases. They show that most of the enzymes can have much higher activities against SPs than they have against OPs. However, just as bioassay data [42], [43] show qualitative differences between the isomers in their insecticidal potencies, these data show large differences between them in their susceptibilities to esteratic break down. Moreover they show that different esterases vary greatly in their isomer preferences and, importantly, that some esterases are relatively active against the most insecticidal 1*(R)trans-α(S)* and 1*(R)cis-α(S)* cypermethrin and 2*(S)-α(S)* fenvalerate (esfenvalerate) isomers.

It was largely the esterases from the smaller Clades 14, 16 and 17 associated with the R_m_ 0.22–0.27 region, rather than the more rapidly migrating Clade 1 esterases, that had the greatest activities for the insecticidal SP isomers. However one Clade 1 esterase, 001g, migrating in the 0.39–0.43 region associated with SP (and OP) resistance, also had relatively high activity against esfenvalerate – this is the SP most commonly used against both the Chinese and Australian populations studied for esterase isozyme-SP resistance associations [9], [21]. Interestingly the Clades 14 and 17 enzymes were predicted to be intracellular while the Clade 16 and 001g enzymes are secreted. Both secreted and intracellular esterases have been linked to OP resistance in various insects [32] and the same may be true for SP resistance.

Coppin *et al.* [20] (and see also the controls in our Table 2) found that substitutions at ten different active site residues of the blowfly E3 esterase could profoundly alter its cypermethrin and fenvalerate isomer preferences. Notably in this respect, 001g was not one of the four Clade 1 esterases which Wu *et al*. [10] used in their study but the four they did use (001a, 001d, 001i and 001j), which had all also shown elevated expression in their resistant strain, were associated with elevated activity against a naphthyl derivative of fenvalerate. However the three of these that we have expressed (001d, 001i and 001j) from a susceptible strain all show relatively little activity against the most insecticidal form of fenvalerate. These differences may reflect either the greater susceptibility of the naphthyl fenvalerate derivative to hydrolysis or genuine differences between the activity spectra of enzymes from susceptible and resistant strains, or both.

Unlike the situation for OP resistance, the relatively high overall activities of the esterases against the SPs and the isomer-specific differences among them argue against a hypothesis that resistance is caused by sequestration via wholesale over-expression of midgut esterases. Hydrolysis seems a much more likely basis for resistance. However, as with the OP resistance, hypotheses that at least some of the resistance is due to esterases that we have not expressed or to greater activities of allelic variants in resistant lines are also viable for the SPs. Further sequencing and biochemical characterisation of midgut esterases, including of the versions found in resistant strains, will be required to resolve these competing hypotheses.

There is good evidence for some low level cross resistance to OPs and SPs in *H. armigera*, and at least some evidence that it may in part be mediated by esterases [9]–[11]. Our results are consistent with this proposition to the extent that we find isozymes such as 001g, 014a and 017a from the relevant zymogram zones which show both tight binding/low activity against OPs and quite high activities against some of the most insecticidal SP isomers tested.

We have empirically confirmed the predictions of Teese *et al*. [21] that three of the *H. armigera* midgut esterases, 001f, 001g and 016a, are GPI-anchored to cell membranes. This is interesting in itself, because no insect esterases other than AChE have previously been shown to have GPI anchors, and our data show that these enzymes have little or no AChE activity. However it is also interesting in the context of the associations between midgut esterase activity and Cry1Ac resistance reported for this species by Young *et al.* [12] and Gunning *et al*. [13], [14]. Teese *et al*. [21] suggested this might be mediated by the binding of the toxin to N-acetylgalactosamine (GalNAc) glycans on GPI-anchored esterases in the midgut, since such glycans are involved in the binding of the toxin to other GPI-anchored midgut proteins known to be targets for the toxin. However we find that none of the three esterases in question sit in the region of the zymogram previously associated with Cry1Ac resistance. The Teese *et al*. [21] hypothesis thus only remains viable if other, as yet undiscovered, esterases possessing the appropriate glycans and GPI-anchors prove to migrate to the relevant zymogram region.

## Supporting Information

Figure S1 **Primers used in the preparation and validation of baculovirus constructs.** Constructs were either synthesised commercially (GeneArt, Germany) with flanking attB extensions and translation initiation sequence included, or PCR amplified from plasmids from a midgut cDNA library [44] using the indicated primers and DNA polymerases with proof-reading capability (Pwo, Roche, USA or Phusion, Finnzymes, Finland).
(DOC)

Figure S2 **Comparison of R_m_ values for seven isozymes expressed herein (E) with zones of activity associated with these isozymes in previous proteomic studies (P).** On the left is shown a slice of a native PAGE gel of a mass homogenate of fifth instar GR larvae stained for esterase activity, as per Figure 3.
(XLSX)

## References

[1] McCaffery AR (1998) Resistance to insecticides in heliothine Lepidoptera: a global view. Phil Trans Roy Soc Lond B 353: 1735–1750.

[2] Kranthi KR, Jadhav DR, Wanjari RR, Shakir Ali S, Russell D (2001) Carbamate and organophosphate resistance in cotton pests in India, 1995 to 1999. Bull Entomol Res 91: 37–46. 11228586

[3] Yang Y-H, Chen S, Wu Y-D (2008) Cross resistance patterns and biochemical mechanisms in a Chinese *Helicoverpa armigera* strain selected with an organophosphate/pyrethroid mixture. Cotton Sci 20: 249–255.

[4] Joußen N, Agnolet S, Lorenz S, Schöne SE, Ellinger R, et al (2012) Resistance of Australian *Helicoverpa armigera* to fenvalerate is due to the chimeric P450 enzyme CYP337B3. Proc Natl Acad Sci USA 109: 15206–15211. 22949643 10.1073/pnas.1202047109 PMC3458352

[5] Gunning RV, Moores GD, Devonshire AL (1996) Esterases and esfenvalerate resistance in Australian *Helicoverpa armigera* (Hübner) Lepidoptera:Noctuidae. Pestic Biochem Physiol 54: 12–23. 10.1006/pest.1996.0031 8980026

[6] Gunning RV, Moores GD, Devonshire AL (1998) Inhibition of resistance-related esterases by piperonyl butoxide in *Helicoverpa armigera* (Lepidoptera: Noctuidae) and *Aphis gossypii* (Hemiptera: Aphididae). In: Jones DG, editor. Piperonyl Butoxide: The Insecticide Synergist. London: Academic Press. 215–226.

[7] Gunning RV, Moores GD, Devonshire AL (1999) Esterase inhibitors synergise the toxicity of pyrethroids in Australian *Helicoverpa armigera* (Hübner) (Lepidoptera: Noctuidae). Pestic Biochem Physiol 63: 50–62. 10.1006/pest.1996.0031 8980026

[8] Achaleke J, Martin T, Ghogomu RT, Vaissayre M, Brévault T (2009) Esterase-mediated resistance to pyrethroids in field populations of *Helicoverpa armigera* (Lepidoptera: Noctuidae) from Central Africa. Pest Manag Sci 65: 1147–1154. 19548293 10.1002/ps.1807

[9] Farnsworth CA, Teese MG, Yuan GR, Li YQ, Scott C, et al (2010) Esterase-based metabolic resistance to insecticides in heliothine and spodopteran pests. J Pestic Sci 35: 275–289.

[10] Wu SW, Yang YH, Yuan GR, Campbell PM, Teese MG, et al (2011) Overexpressed esterases in a fenvalerate resistant strain of the cotton bollworm, *Helicoverpa armigera* . Insect Biochem Mol Biol 41: 14–21. 20875855 10.1016/j.ibmb.2010.09.007

[11] Han Y, Wu S, Li Y, Liu J-W, Campbell PM, et al (2012) Proteomic and molecular analyses of esterases associated with monocrotophos resistance in *Helicoverpa armigera* . Pestic Biochem Physiol 104: 243–251.

[12] Young SJ, Gunning RV, Moores GD (2005) The effect of piperonyl butoxide on pyrethroid-resistance-associated esterases in *Helicoverpa armigera* Hübner Lepidoptera: Noctuidae. Pest Manag Sci 61: 397–401. 15605351 10.1002/ps.996

[13] Gunning RV, Dang HT, Kemp FC, Nicholson IC, Moores GD (2005) New resistance mechanism in *Helicoverpa armigera* threatens transgenic crops expressing *Bacillus thuringiensis* Cry1Ac toxin. Appl Environ Microbiol 71: 2558–2563. 15870346 10.1128/AEM.71.5.2558-2563.2005 PMC1087549

[14] Gunning RV, Nicholson IC, Kemp FC, Borzatta V, Cottage E, et al (2006) Piperonyl butoxide, restores the efficacy of *Bacillus thuringiensis* toxin in transgenic cotton against resistant *Helicoverpa armigera* . Biopest Internat 2: 129–136.

[15] Alvi AHK, Sayyed AH, Naeem M, Ali M (2012) Field evolved resistance in *Helicoverpa armigera* (Lepidoptera: Noctuidae) to *Bacillus thuringiensis* toxin Cry1Ac in Pakistan. PLoS ONE 7: e47309. 23077589 10.1371/journal.pone.0047309 PMC3471837

[16] Li X, Schuler MA, Berenbaum MR (2007) Molecular mechanisms of metabolic resistance to synthetic and natural xenobiotics. Annu Rev Entomol 52: 231–253. 16925478 10.1146/annurev.ento.51.110104.151104

[17] Bass C, Field LM (2011) Gene amplification and insecticide resistance. Pest Manag Sci 67: 886–890. 21538802 10.1002/ps.2189

[18] Heidari R, Devonshire AL, Campbell BE, Dorrian SJ, Oakeshott JG, et al (2005) Hydrolysis of pyrethroids by carboxylesterases from *Lucilia cuprina* and *Drosophila melanogaster* with active sites modified by *in vitro* mutagenesis. Insect Biochem Mol Biol 35: 597–609. 15857765 10.1016/j.ibmb.2005.02.018

[19] Devonshire AL, Heidari R, Huang HZ, Hammock BD, Russell RJ, et al (2007) Hydrolysis of individual isomers of fluorogenic pyrethroid analogs by mutant carboxylesterases from *Lucilia cuprina* . Insect Biochem Mol Biol 37: 891–902. 17681228 10.1016/j.ibmb.2007.04.011

[20] Coppin CW, Jackson CJ, Sutherland T, Hart PJ, Devonshire AL, et al (2012) Testing the evolvability of an insect carboxylesterase for the detoxification of synthetic pyrethroid insecticides. Insect Biochem Mol Biol 42: 343–352. 22300675 10.1016/j.ibmb.2012.01.004

[21] Teese MG, Campbell PM, Scott C, Gordon KHJ, Southon A, et al (2010) Gene identification and proteomic analysis of the esterases of the cotton bollworm, *Helicoverpa armigera* . Insect Biochem Mol Biol 40: 1–16. 20005949 10.1016/j.ibmb.2009.12.002

[22] Heidari R, Devonshire AL, Campbell BE, Bell KL, Dorrian SJ, et al (2004) Hydrolysis of organophosphorus insecticides by *in vitro* modified carboxylesterase E3 from *Lucilia cuprina* . Insect Biochem Mol Biol 34: 353–363. 15041019 10.1016/j.ibmb.2004.01.001

[23] Devonshire AL, Heidari R, Bell KL, Campbell PM, Campbell BE, et al (2003) Kinetic efficiency of mutant carboxylesterases implicated in organophosphate insecticide resistance. Pestic Biochem Physiol 76: 1–13.

[24] Incardona JP, Rosenberry TL (1996) Construction and characterization of secreted and chimeric transmembrane forms of *Drosophila* acetylcholinesterase: a large truncation of the C-terminal signal peptide does not eliminate glycoinositol phospholipid anchoring. Mol Biol Cell 7: 595–611. 8730102 10.1091/mbc.7.4.595 PMC275912

[25] Li W, Pi R, Chan HH, Fu H, Lee NT, et al (2005) Novel dimeric acetylcholinesterase inhibitor bis7-tacrine, but not donepezil, prevents glutamate-induced neuronal apoptosis by blocking N-methyl-D-aspartate receptors. J Biol Chem 280: 18179–18188. 15710623 10.1074/jbc.M411085200

[26] Mastropaolo W, Yourno J (1981) An ultraviolet spectrophotometric assay for α-naphthyl acetate and α-naphthyl butyrate esterases. Anal Biochem 115: 188–193. 7304943 10.1016/0003-2697(81)90544-3

[27] Ellman GL, Courtney KD, Andres Jr V, Featherstone RM (1961) A new and rapid colorimetric determination of acetylcholinesterase activity. Biochem Pharmacol 7: 88–95. 13726518 10.1016/0006-2952(61)90145-9

[28] Moores GD, Devine GJ, Devonshire AL (1994) Insecticide-insensitive acetylcholinesterase can enhance esterase-based resistance in *Myzus persicae* and *Myzus nicotianae* . Pestic Biochem Physiol 49: 114–120.

[29] Washington MT, Prakash L, Prakash S (2003) Mechanism of nucleotide incorporation opposite a thymine-thymine dimer by yeast DNA polymerase η. Proc Natl Acad Sci USA 100: 12093–12098. 14527996 10.1073/pnas.2134223100 PMC218718

[30] Tsubota T, Shimomura M, Ogura T, Seino A, Nakakura T, et al (2010) Molecular characterization and functional analysis of novel carboxyl/cholinesterases with GQSAG motif in the silkworm *Bombyx mori* . Insect Biochem Mol Biol 40: 100–112. 20060470 10.1016/j.ibmb.2009.12.015

[31] Myers M, Healy M, Oakeshott J (1996) Mutational analysis of *N*-linked glycosylation of esterase 6 in *Drosophila melanogaster* . Biochem Genet 34: 201–218. 8813053 10.1007/BF02407020

[32] Oakeshott JG, Claudianos C, Campbell PM, Newcomb RD, Russell RJ (2005) Biochemical genetics and genomics of insect esterases. In: Gilbert LI, Iatrou K, Gill SS, editors. Comprehensive Molecular Insect Science. London: Elsevier. 309–361.

[33] Oakeshott JG, Papenrecht EA, Boyce TM, Healy MJ, Russell RJ (1993) Evolutionary genetics of *Drosophila* esterases. Genetica 90: 239–268. 8119594 10.1007/BF01435043

[34] Massoulié J, Anselmet A, Bon S, Krejci E, Legay C, et al (1999) The polymorphism of acetylcholinesterase: post-translational processing, quaternary associations and localization. Chem-Biol Interact 119–120: 29–42. 10.1016/s0009-2797(99)00011-3 10421436

[35] Horejsi T, Box JM, Staub JE (1999) Efficiency of Randomly Amplified Polymorphic DNA to sequence characterized amplified region marker conversion and their comparative Polymerase Chain Reaction sensitivity in cucumber. J Am Soc Hort Sci 124: 128–135.

[36] Campbell BE (2001) The role of esterases in Australian populations of the cotton bollworm, *Helicoverpa armigera* (Hübner) (Lepidoptera: Noctuidae) [PhD]. Canberra: Australian National University.

[37] Campbell PM, Newcomb RD, Russell RJ, Oakeshott JG (1998) Two different amino acid substitutions in the ali-esterase, E3, confer alternative types of organophosphorus insecticide resistance in the sheep blowfly, *Lucilia cuprina* . Insect Biochem Mol Biol 28: 139–150.

[38] Hartley CJ, Newcomb RD, Russell RJ, Yong CG, Stevens JR, et al (2006) Amplification of DNA from preserved specimens shows blowflies were preadapted for the rapid evolution of insecticide resistance. Proc Natl Acad Sci USA 103: 8757–8762. 16723400 10.1073/pnas.0509590103 PMC1482651

[39] Cui F, Lin Z, Wang H, Liu S, Chang H, et al (2011) Two single mutations commonly cause qualitative change of nonspecific carboxylesterases in insects. Insect Biochem Mol Biol 41: 1–8. 20888910 10.1016/j.ibmb.2010.09.004

[40] Heidari R (2002) Kinetic properties of esterases hydrolysing organophosphorus and carboxylester insecticides [PhD]: Charles Sturt University.

[41] Jackson C, Sanchez-Hernandez J, Wheelock C, Oakeshott J (2010) Carboxylesterases in the metabolism and toxicity of pesticides. In: Satoh T, Gupta R, editors. Anticholinesterase Pesticides: Metabolism, Neurotoxicity and Epidemiology. Hoboken: John Wily & Sons. 57–76.

[42] Elliott M, Farnham AW, Janes NF, Needham PH, Pulman DA (1974) Insecticidally active conformations of pyrethroids. In: Kohn GK, editor. Mechanisms of Pesticide Action. Washington D.C.: American Chemical Society. 80–91.

[43] Pap L, Kelemen M, Tóth A, Székely I, Bertók B (1996) The synthetic pyrethroid isomers II. Biological activity. J Environ Sci Health Part B: Pestic Food Contam Agric Wastes 31: 527–543.

[44] Angelucci C, Barrett-Wilt GA, Hunt DF, Akhurst RJ, East PD, et al (2008) Diversity of aminopeptidases, derived from four lepidopteran gene duplications, and polycalins expressed in the midgut of *Helicoverpa armigera*: Identification of proteins binding the δ-endotoxin, Cry1Ac of *Bacillus thuringiensis* . Insect Biochem Mol Biol 38: 685–696. 18549954 10.1016/j.ibmb.2008.03.010 PMC2852237

