# Supplementary material for: Heterologous Expression and Biochemical Characterisation of Fourteen Esterases from Helicoverpa armigera
Source: PLoS One. 2013 Jun 17;8(6):e65951. doi: 10.1371/journal.pone.0065951 (PMC3684599; doi:10.1371/journal.pone.0065951)
Supplement: Figure S1 — Primers used in the preparation and validation of baculovirus constructs. Constructs were either synthesised commercially (GeneArt, Germany) with flanking attB extensions and translation initiation sequence included, or PCR amplified from plasmids from a midgut cDNA library [44] using the indicated primers and DNA polymerases with proof-reading capability (Pwo, Roche, USA or Phusion, Finnzymes, Finland). (DOC) [file pone.0065951.s001.doc]

**Supplementary Figure 1**

| **Gene** | **Accession** | **Origin*** | **Primer Name** | **Primer sequence** |
| --- | --- | --- | --- | --- |
| eGFP | U55761 | PCR | attB1-eGFP | GGGGACAAGTTTGTACAAAAAAGCAGGCTTATTCAAAATGGTGAGCAAGGGCGAGGAGCTG |
|  |  |  | attB2-eGFP | GGGGACCACTTTGTACAAGAAAGCTGGGTAAGGTTCAGGGGGAGGTGTGGGAGG |
|  |  |  |  |  |
| 001b ^†^ | KC589105 | PCR | attB1-Hax041-F05 | GGGGACAAGTTTGTACAAAAAAGCAGGCTTATTCAAAATGAAGTGGTGGACGTGTGTGGTGTTCATGTGCGCG |
|  |  |  | attB2-Hax041-R05 | GGGGACCACTTTGTACAAGAAAGCTGGGTATCCTACTACTTCTACAGCTCGTCTCGTGGTCTGGGC |
|  |  |  |  |  |
| 001c | FJ997294 | PCR | attB1-001c | GGGGACAAGTTTGTACAAAAAAGCAGGCTTATTCAAAATGATGAAGTGGTGGACGTGTG |
|  |  |  | attB2-001c | GGGGACCACTTTGTACAAGAAAGCTGGGTAATTTATTTGAGAATTATATTTCCACGCTACG |
|  |  |  |  |  |
| 001d | FJ997295 | Synthesis |  |  |
|  |  |  |  |  |
| 001f ^†^ | FJ997297 | PCR | attB1-LongHax043-F02 | GGGGACAAGTTTGTACAAAAAAGCAGGCTTATTCAAAATGTGGTGGCGCACGTGTGTGGTGCTGGTCTGCGTGA |
|  |  |  | attB2-LongHax043-R02 | GGGGACCACTTTGTACAAGAAAGCTGGGTATTCTGGTTGCAAAACTAAGGGTATCAAAGC |
|  |  |  |  |  |
| 001g | KC589104 | PCR | attB1-Hax044-F01 | GGGGACAAGTTTGTACAAAAAAGCAGGCTTATTCAAAATGTGGTGGCGCACGTGTGT |
|  |  |  | attB2-Hax044-R01 | GGGGACCACTTTGTACAAGAAAGCTGGGTACGGGTCGGCCTAATAGAAGCATG |
|  |  |  |  |  |
| 001h | FJ997299 | PCR | attB1-Hax045-F01 | GGGGACAAGTTTGTACAAAAAAGCAGGCTTATTCAAAATGAGGCGACTGTTGATAGTGTTA |
|  |  |  | attB2-Hax045-R01 | GGGGACCACTTTGTACAAGAAAGCTGGGTACGGACAATGGTGTAACCTCGT |
|  |  |  |  |  |
| 001i | FJ997302 | PCR | attB1-001i | GGGGACAAGTTTGTACAAAAAAGCAGGCTTATTCAAAATGAAAATAATGTATTTGGTGACGA |
|  |  |  | attB2-001i | GGGGACCACTTTGTACAAGAAAGCTGGGTACAACTATGCACATACTTATTTCTGCCG |
|  |  |  |  |  |
| 001j | JQ684436 | PCR | attB1-Hax039-F01 | GGGGACAAGTTTGTACAAAAAAGCAGGCTTATTCAAAATGAAGTGGTGGACGTGTGTGG |
|  |  |  | attB2-Hax039-R01 | GGGGACCACTTTGTACAAGAAAGCTGGGTATTACAACTCGTTGCGTGGTCTGGGCG |
|  |  |  |  |  |
| 006a | FJ997304 | PCR | attB1-006a | GGGGACAAGTTTGTACAAAAAAGCAGGCTTATTCAAAATGAACATCATGGGGGACAAAATCATATTC |
|  |  |  | attB2-006a | GGGGACCACTTTGTACAAGAAAGCTGGGTACACCAGCCTAAGAAGAGTACGAGTAAGCTC |
|  |  |  |  |  |
| 006b | FJ997306 | PCR | attB1-006b | GGGGACAAGTTTGTACAAAAAAGCAGGCTTATTCAAAATGTCTAAATGCTTGGACATGTTGTCTGTTCTAACACTAGTGTTATTATGTGGAT |
|  |  |  | attB2-006b | GGGGACCACTTTGTACAAGAAAGCTGGGTATTGACTCCGCTTTACCTACCAATGAAG |
|  |  |  |  |  |
| 014a | FJ997310 | Synthesis |  |  |
|  |  |  |  |  |
| 016a | FJ997313 | PCR | attB1-016a | GGGGACAAGTTTGTACAAAAAAGCAGGCTTATTCAAAATGACGTCAGTGATACATATCGTGTC |
|  |  |  | attB2-016a | GGGGACCACTTTGTACAAGAAAGCTGGGTAGTAATCAATCAGTACAGTCGAGGCTTTGG |
|  |  |  |  |  |
| 017a | FJ997318 | PCR | attB1-017a | GGGGACAAGTTTGTACAAAAAAGCAGGCTTATTCAAAATGTTTGTTATAAGAACTATTGAAAAG |
|  |  |  | attB2-017a | GGGGACCACTTTGTACAAGAAAGCTGGGTATAAACGACATATCCCCAAATAGCCAG |
|  |  |  |  |  |
| 025a | FJ997326 | PCR | attB1-025a | GGGGACAAGTTTGTACAAAAAAGCAGGCTTATTCAAAATGCCAAACAGGTTTCATTTAAAAAGCGTG |
|  |  |  | attB2-025a | GGGGACCACTTTGTACAAGAAAGCTGGGTATTATAGTTCATCCATAAACCTACTATTACG |

* Constructs were either synthesised commercially (GeneArt, Germany) with flanking attB extensions and translation initiation sequence included, or PCR amplified from plasmids from a midgut cDNA library (Angelucci et al. 2008) using the indicated primers and DNA polymerases with proof-reading capability (Pwo, Roche, USA or Phusion, Finnzymes, Finland). Constructs were cloned into pDONR201 to form the entry vectors (pENTR201-GOI), using the Gateway BP reaction as per standard Gateway protocols (Invitrogen, USA). Entry vectors were sequenced using pDONR201 or gene‑specific primers to confirm that they contained the correct insert. The LR reaction was used to transfer the gene of interest into linear *Autographa californica* nucleopolyhedrosis virus (AcNPV) DNA.

^†^ Sequences based on near complete cDNA sequences augmented with sequence data from the unpublished *H. armigera* genome.

Black attB1 sequence; Grey attB2 sequence; TTCAAA *H. armigera* actin translation initiation sequence (GenBank accession no. X97615); Underlined Gene specific sequence.
